# A new versatile MR-guided high-intensity focused ultrasound (HIFU) device for the treatment of musculoskeletal tumors

**DOI:** 10.1038/s41598-022-13213-1

**Published:** 2022-05-31

**Authors:** Paolo Cabras, Pierre Auloge, Fabrice Bing, Pramod Prabhakar Rao, Stéphanie Hoarau, Erik Dumont, Alexandre Durand, Benjamin Maurin, Benoit Wach, Loïc Cuvillon, Elodie Breton, Afshin Gangi, Jonathan Vappou

**Affiliations:** 1 ICube, Université de Strasbourg, CNRS, UMR 7357, Strasbourg, France; 2 Image Guided Therapy, Pessac, France; 3 Department of Interventional Imaging, Hôpitaux Universitaires de Strasbourg, Strasbourg, France; 4 Radiology Department, Hôpital d’Annecy, Metz-Tessy, France; 5 Axilum Robotics, Schiltigheim, France

**Keywords:** Biomedical engineering, Bone cancer, Bone metastases, Cancer therapy

## Abstract

Magnetic Resonance (MR) Imaging-guided High Intensity focused Ultrasound (MRgHIFU) is a non-invasive, non-ionizing thermal ablation therapy that is particularly interesting for the palliative or curative treatment of musculoskeletal tumors. We introduce a new modular MRgHIFU device that allows the ultrasound transducer to be positioned precisely and interactively over the body part to be treated. A flexible, MR-compatible supporting structure allows free positioning of the transducer under MRI/optical fusion imaging guidance. The same structure can be rigidified using pneumatic depression, holding the transducer rigidly in place. Targeting accuracy was first evaluated in vitro. The average targeting error of the complete process was found to be equal to 5.4 ± 2.2 mm in terms of focus position, and 4.7° ± 2° in terms of transducer orientation. First-in-man feasibility is demonstrated on a patient suffering from important, uncontrolled pain from a bone metastasis located in the forearm. The 81 × 47 × 34 mm^3^ lesion was successfully treated using five successive positions of the transducer, under real-time monitoring by MR Thermometry. Significant pain palliation was observed 3 days after the intervention. The system described and characterized in this study is a particularly interesting modular, low-cost MRgHIFU device for musculoskeletal tumor therapy.

## Introduction

High Intensity Focused Ultrasound (HIFU) therapy is a non-invasive, non-ionizing, ablation method that relies on the localized absorption of the acoustic energy^1^. As a non-invasive method, HIFU therapy requires medical imaging for procedure planning and targeting, therapy monitoring in real time and post operative assessment of the intervention. Magnetic Resonance Imaging (MRI) is considered as the gold standard method for controlling HIFU thermal ablations in real time, thanks to its ability to provide tissue temperature in real time through MR Thermometry^2–5^. MR-guided HIFU (MRgHIFU) is currently used clinically for several applications such as the treatment of uterine fibroids^6,7^ or the treatment of essential tremor^8,9^.

MRgHIFU has also been proposed as a palliative or curative method for treating musculoskeletal tumors, such as bone metastases^10–15^, osteoid osteomas^16–18^, or desmoid tumors^19,20^. MRgHIFU is indeed particularly promising in bone, because the high acoustic absorption of bone tissue^21,22^ leads to high temperature elevation^23^, even at low acoustic power. The clinical significance of MRgHIFU is particularly high in the case of bone metastases, whose palliative treatment represents a major medical challenge. Lengthening of life expectancy and progress in treatment of primary cancers lead to a high number of patients that must live with often painful, debilitating bone metastases^24,25^. Common therapeutic strategies such as radiotherapy and opioid therapy exhibit significant limitations in terms of efficiency, repeatability, and patient quality of life^26^. In this context, MRgHIFU has emerged as a non-invasive, non-ionizing, repeatable alternative for palliative treatment of bone metastases. In the case of benign bone tumors such as osteoid osteoma, resection surgery and/or percutaneous ablation are the clinical gold standard^27,28^. For this indication, the benefit of MRgHIFU is mostly its non-invasive nature.

Most studies reporting the use of MRgHIFU for musculoskeletal tumors have been carried out with “in-table transducer” whole body MRI systems that were initially developed for the treatment of uterine fibroids. In such a configuration, the patient lies on a customized MR-table containing the HIFU transducer in a water bath. The patient is positioned so that the region to be treated lies on the acoustic window of the MR table. Hence the patient must often lie on a potentially painful body site, which is an obstacle for procedure planning^29^. Such devices can be adapted with relative ease for the treatment of bone tumors located in the pelvic area, granted that the patient can be positioned with the adequate orientation. Patient positioning and/or accessibility to the target can become much more challenging or even impossible for lesions located in other body parts. From a purely technical point-of-view, such systems were not developed for this clinical indication, and they meet only partially the specifications of the ideal MRgHIFU system for treating musculoskeletal tumors. The ideal MRgHIFU device for the treatment of musculoskeletal tumors should allow the transducer to be positioned over the patient, regardless of patient position, rather than the opposite. It should be modular and adaptable to different parts of the body. Ideally, it should also allow high flexibility in terms of HIFU transducer properties, such as acoustic aperture, focal length and ultrasonic central frequency. For example, osteoblastic and osteolytic tissues greatly differ in terms of acoustic properties and using different ultrasonic frequencies and focal lengths could be relevant.

In this paper, we introduce a novel MRgHIFU device with a “transducer on patient” approach, to significantly increase the accessibility to different parts of the body. This system relies on the following main features: (1) The physician should be able to manipulate the HIFU transducer freely with 6 degrees of freedom, in a similar fashion as what he/she does when positioning a needle before a percutaneous intervention; (2) During this positioning phase, the physician should be able to see the position of the transducer in real time with respect to the preoperative image and the target; (3) The transducer position and orientation should be fixed and locked once the physician estimates the transducer is positioned correctly; (4) The physician can choose the most adequate transducer in terms of acoustic properties. This device has several important, obvious specifications such as MR compatibility, acoustic coupling without air between the transducer and the patient, and compliance with regulatory requirements for its clinical use. In this paper, we demonstrate that the HIFU transducer can be positioned accurately to its planned position, and we report a first clinical feasibility study of the palliative treatment of a bone metastasis located in the forearm.

## Materials and methods

### System description

#### Overview

The proposed HIFU device relies on three main units: the preoperative planning unit, the transducer positioning unit, and the HIFU unit.

- The preoperative planning unit is a software interface that allows the physician to position the transducer virtually on preoperative images. By visualizing the corresponding ultrasonic cone, he/she can decide the best transducer pose (position and orientation) that should be used during the intervention.
- The positioning unit is composed of a semi-flexible structure holding the transducer and coupled with a navigation software. It assists the physician in positioning the transducer to the preoperatively planned position, and then maintains the transducer in place during the procedure.
- The HIFU unit is composed of a 256-channel generator, a multi-element HIFU transducer with its water-filled balloon acoustic coupling system, and the corresponding software user-interface for driving the generator and displaying real-time MR thermometry images for therapy monitoring.

#### Planning unit

Planning is fundamental to establish the best strategy for the intervention and to mitigate the associated risks. Specifically, for HIFU, the possible interaction of the ultrasound waves with tissues ahead and beyond the focus in the ultrasonic path must be anticipated to minimize unwanted effects.

**Figure 1:**
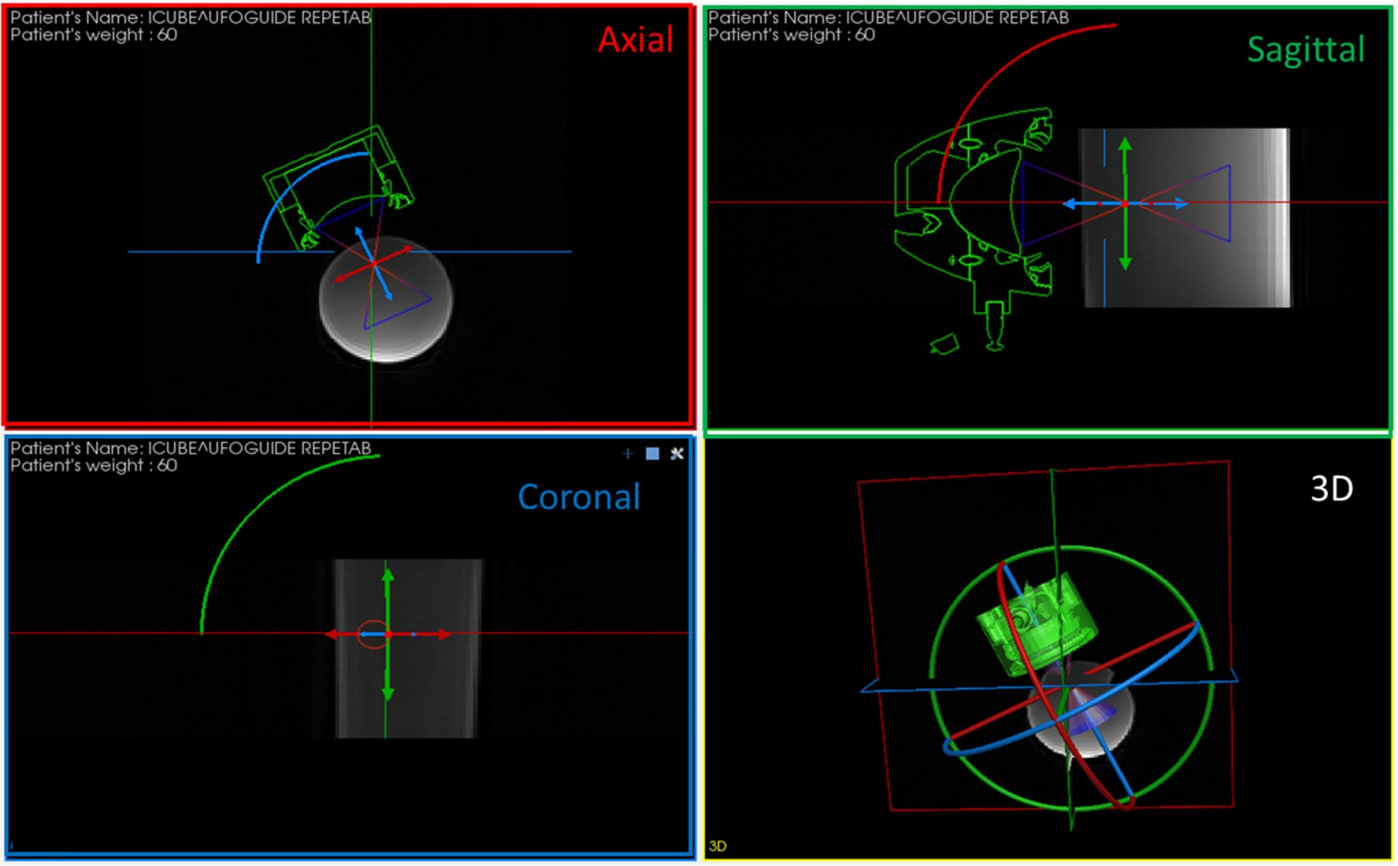
Preoperative planning interface illustrated on MR images of a uniform phantom. The practitioner can rotate and translate the ultrasonic transducer virtually in all imaging planes. By visualizing the anatomical structures exposed to the ultrasonic beam, the practitioner can choose the optimal transducer poses, which can be saved for the time of the intervention. Colored arcs represent the transducer pathway when performing a rotation around the focus.

A specific software has been designed and developed using Qt (Qt Group PIc, Helsinki, Finland) for the user interface and the MITK library (German Cancer Research Center (DKFZ), Heidelberg, Germany) for visualizing and navigating within MR volumetric datasets. This software allows measuring distances, computing possible collisions between the transducer and the body of the patient as well as saving and reloading transducer poses that should be used during the intervention. The virtual transducer is interactively positioned directly on the patient preoperative image volume (Fig. 1) through a 3D draggable object anchored to the 3D transducer model.

The ultrasound field is represented as a cone. The practitioner can easily see which anatomical structures are exposed to the ultrasound beam thanks to the visualization of this cone attached to the transducer, as well as the corresponding cone sections on the reconstructed planes. The visualization of the image plane perpendicular to the US cone is available as an option. In such a plane, the section of the ultrasound cone is a circle, and the user can verify whether sensitive structures are located in or near the circle.

#### Positioning unit: transducer holding structure

**Figure 2:**
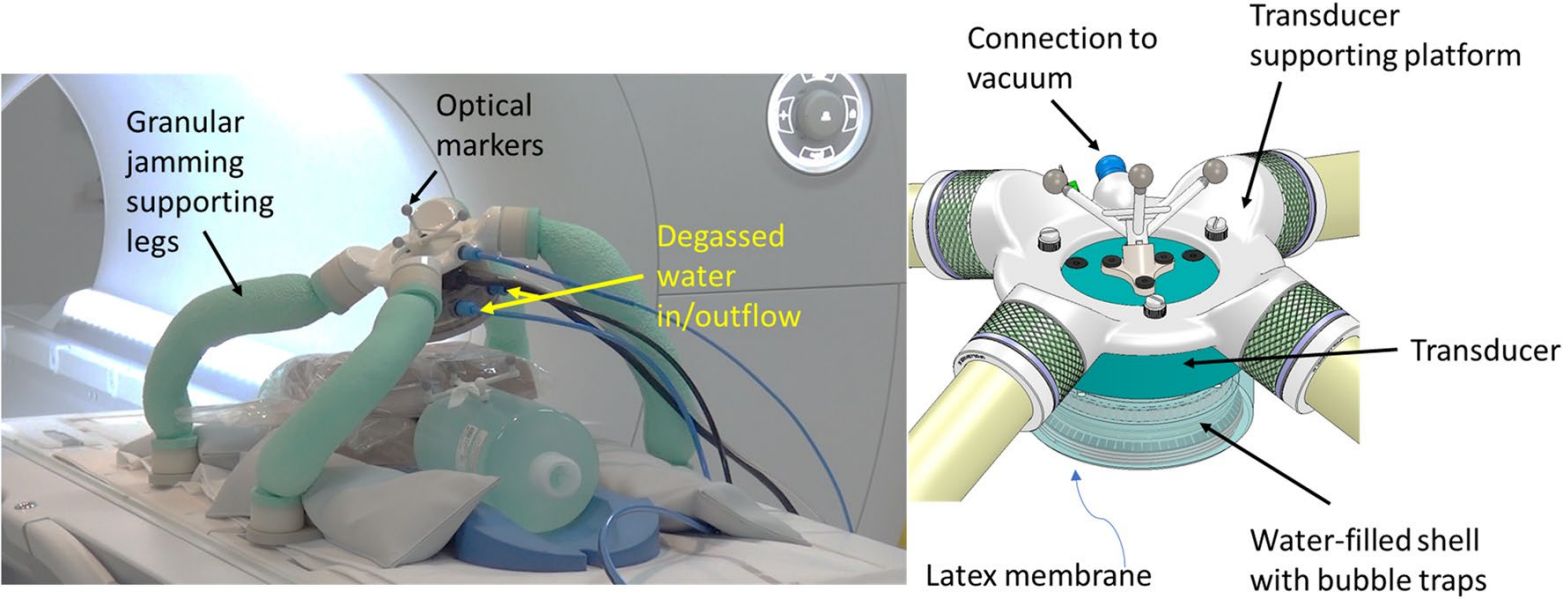
Photograph and schematic view of the holding system. The ultrasound transducer is encapsulated in a water-filled shell and fixed to a transducer-specific supporting platform. This platform is hermetically connected to the granular jamming supporting legs, which are naturally flexible and can be rigidified by applying a pneumatic depression.

As explained in the previous section, whereas a “transducer on-patient” architecture offers the possibility to extend the MRgHIFU therapy to several body parts, it involves several major technological challenges. Indeed, the system should be able to maintain the transducer position as well as the acoustic contact throughout the whole procedure, it must be MR-compatible and sufficiently compact to fit within the restricted MR tunnel space. The solution proposed here stems from requirements expressed by a large panel of experienced interventional radiologists: The practitioner should be able to move the transducer freely in space with 6 degrees of freedom without specific constraints (forced rotations/translations), while keeping a visual information on the acoustic coupling between the membrane and patient’s skin. Once in the optimal position, the transducer should be maintained in place in a user-friendly manner. The resulting solution consists in a passive platform standing on four legs that can be rigidified using the granular jamming principle^30^: an elastic chamber filled with granular elements can be rigidified when air is removed from the chamber. As shown in Fig. 2, the whole system is composed of one transducer shell, one supporting platform and four legs. The legs are made of 20 µm thick latex, hermetically sealed and filled by irregular granular elements (polypropylene beads, 4 mm diameter). The legs are rigidified through air aspiration (hospital medical vacuum plug or portable pump) connected to the platform vacuum connector. Each leg is terminated with a foot orientable in any direction with respect to the leg. Feet can be directly positioned under the patient’s body or ballasted with sandbags. The transducer holding structure can also operate with two or three legs, provided that the remaining leg connectors are sealed on the transducer supporting platform.

The diameter and density of the legs were empirically determined to sustain more than twice the load of the transducer with its shell fully filled with water, which weights around 760 g. The transducer is encapsulated in a shell made of rigid plastic and a latex membrane and filled with degassed water for acoustic transmission of the ultrasound waves between the transducer and the patient. The latex membrane is mounted on a rigid ring, forming a disposable piece that is screwed to the rigid part of the shell. The architecture of the shell includes the presence of bubble traps to protect the active surface of the transducer from air bubbles, as well as special ducts that allow evacuating these bubbles during the water filling process. The shell also houses three MR-visible markers whose relative pose (with respect to the transducer) is known, allowing for the computation of the transducer pose in the MR reference frame. Finally, the transducer shell is attached to the transducer-supporting platform to which the legs are hermetically fixed.

#### Positioning unit: navigation software

**Figure 3:**
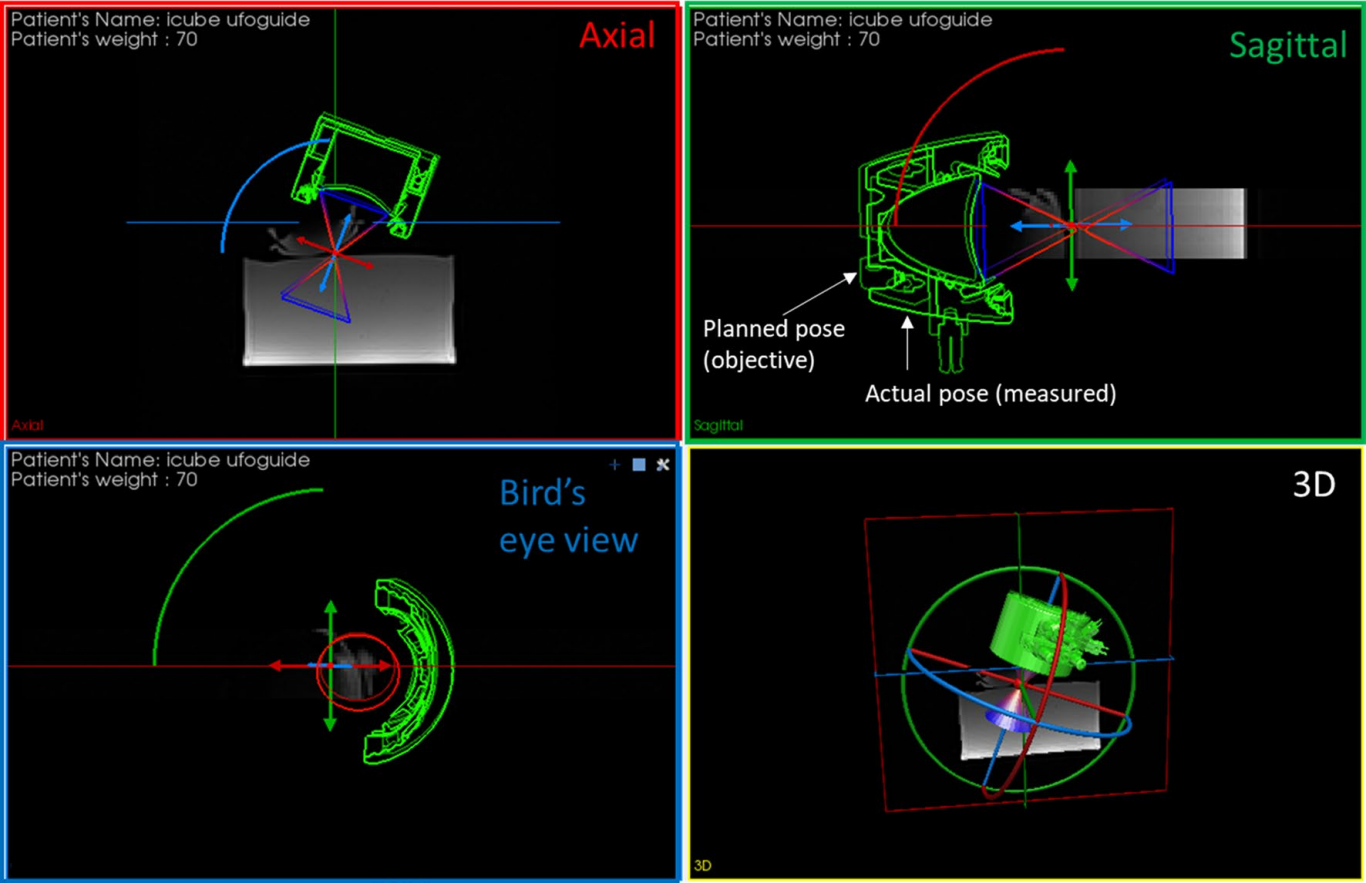
Navigation scene with the actual transducer (plain) close to the planned transducer pose (in transparency). The objective for the practitioner is to move the transducer until the two poses coincide.

Because of the non-invasive nature of the therapy, the physician can obviously not see the target nor the ultrasound cone. A navigation software module has been developed to guide the practitioner during the positioning process. The basic idea underneath this module is to propose a virtual environment where the clinician can see the per-operative patient image, the planned configuration and a model showing—in real time—the actual pose of the transducer he/she is manipulating with respect to the planned pose. The objective is to position the transducer till the virtual transducer model overlaps the planned transducer outline (Fig. 3).

**Figure 4:**
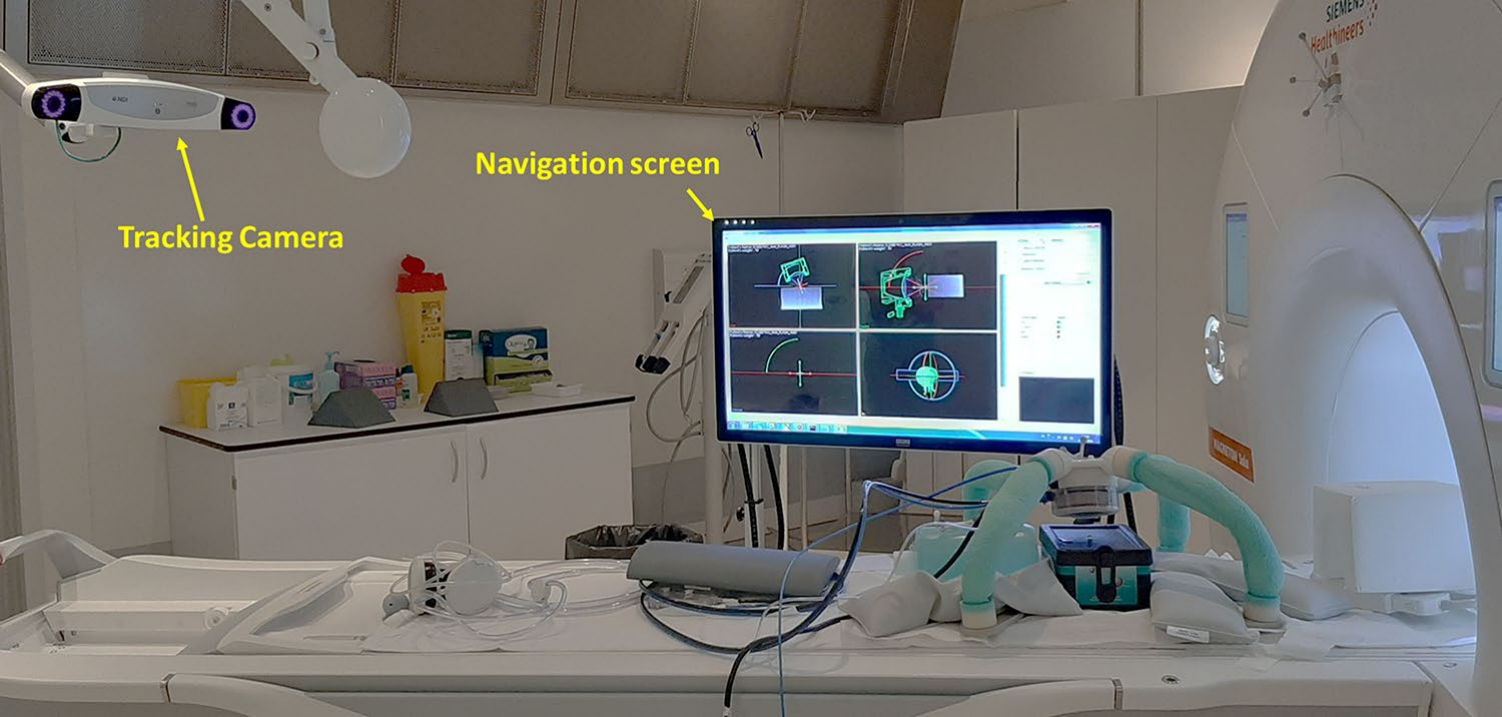
Photograph of the MR room setting.

A commercial near infrared (NIR) stereo-vision system (Polaris Vega, NDI, Canada, Fig. 4) is used for measuring the pose (position + orientation) of all the elements of the scene. This device allows computing, in real time, the poses of specific trackers made of NIR-reflective spheres arranged in a known, rigid geometry. These trackers are attached rigidly to instruments or objects to be tracked.

Since the anatomical image and the transducer's actual pose are expressed with respect to different reference systems (respectively the MR and the Polaris frames), a registration process is required to express all the poses in the same reference frame (also called intraoperative coordinate system).

The registration workflow used here follows a common navigation framework^31^. It relies on an ad hoc tracker visible in both modalities (namely NIR optical and MRI). This *hybrid* tracker is composed of four NIR- reflective spheres (visible to the Polaris camera) filled with a gadolinium-containing solution (acting as landmarks in the MR images). The pose of the hybrid tracker is given directly by the Polaris system, whereas the coordinates of the MR landmarks are obtained interactively: the physician is asked to mark the center of the spheres on an MR image acquired specifically for this process. The hybrid tracker shall be placed ideally near the region to be treated. More details about the specific registration process can be found in the Supplementary Material B.

The registration process allows obtaining the transform between MR to Polaris frames. As a result, the transducer pose can be correctly updated in the virtual scene whose reference frame is the MR image frame.

The MR-table is sled outside the MR-scanner during the registration and positioning process since the markers must lie within the line of sight of the stereo vision system.

#### Positioning protocol

This positioning protocol can be summarized as follows:

1. The patient is prepared, the hybrid tracker is positioned near to the region to be treated and an MR anatomical image is acquired.
2. The patient 3D volume images are loaded in the planning software.
3. A transducer pose is chosen and saved as the desired pose.
4. The patient is sled out the MR tunnel and the registration procedure is performed, as described previously.
5. The user positions the transducer using the navigation system outside the MR tunnel (Fig. 4), following the protocol described in the previous sections “Positioning Unit”. Vacuum is applied and the supporting legs are rigidified once the user considers that the transducer is at the desired position. The MR table is sled to the isocenter of the MRI.
6. Two MR-based verification steps are performed:
  - The first verification step is image and model- based. Anatomical images are acquired. Using the real-time monitoring software (ThermoGuide^32^, Image Guided Therapy, Pessac, France), the 3D coordinates of the MR markers contained in the shell are used to compute the transducer pose. A representation of the ultrasound beam is displayed directly on the anatomical images, showing the actual position of the cone. The practitioner can therefore decide whether the positioning is satisfactory.
  - The second verification step consists in applying a mild, non-harmful hyperthermia (ΔT < 5 °C) using low intensity, low-duration ultrasonic shots. MR thermometry is used to verify that temperature increases only at the desired target.

The therapeutic HIFU ablation is started only after these two verification steps are considered as satisfactory by the practitioners.

#### HIFU unit: treatment and monitoring

The treatment is achieved by exciting the transducer piezoelectric elements with a proper electrical signal. The HIFU transducer is driven by a 256-channel generator (IGT, Pessac, France). The proposed device is compatible with a large spectrum of ultrasound transducers with a maximum of 256 elements. Transducer parameters such as size, shape, number of elements and central frequency can therefore be chosen optimally according to the intended indication, as it will be illustrated below in our first-in-man trial. Each HIFU shot is defined by a duration, a power, a duty cycle and a position in space (defined with respect to the natural reference frame of the transducer). At the same time, it is essential to monitor the temperature in real time to guarantee treatment efficacy and safety. These two features (definition of acoustic trajectories and real-time temperature monitoring) are centralized within the same software (Thermoguide, Image Guided Therapy, Pessac, France). This software allows the user to define the desired acoustic trajectory and to start/stop the HIFU shots. The software controls the signal generator hardware to generate the desired trajectory. Thermoguide retrieves magnitude and phase MR images from the MR scanner in real time and can process those images to compute the temperature. In the present work, temperature was estimated using proton resonance frequency shift^4,5,33^.

### Assessment of mechanical robustness and accuracy

The structural robustness of the holding device and its positioning accuracy were evaluated following two procedures.

#### Mechanical robustness

This first procedure aimed at quantifying a possible drift in the pose of the transducer-holding platform when sustaining a 2 kg load. The pose (i.e. the position and the orientation) of the platform was measured for 45 min with the same stereo vision tracking system used in the navigation unit. Two holding configurations were studied, namely straight and centered arc-like, and strongly inclined (Fig. S1a,b in Supplementary Material A). Such experiments were considered as stress tests, since this weight represents more than twice the weight that the holding system is supposed to carry. Moreover, in real conditions of use, part of the weight of the transducer and its filled shell will be supported by the body of the patient, since the coupling balloon must stay in contact with the skin.

#### Targeting accuracy

The second procedure aimed at assessing the accuracy of the positioning protocol using the computer-aided positioning unit. A trained user was asked to replicate 10 pre-established planned poses using the proposed navigation and holding system. A spherical MR visible marker (Pinpoint, Beekley medical, Bristol, USA, 6 mm in diameter) was attached to a water bottle to serve as the desired target. For all 10 planned poses, the natural focus of the transducer was positioned on that same target, but with different orientations (with a maximum inclination of 30° with respect to the vertical axis), in order to test the accuracy of the device in several representative configurations spanning the system workspace.

**Figure 5:**
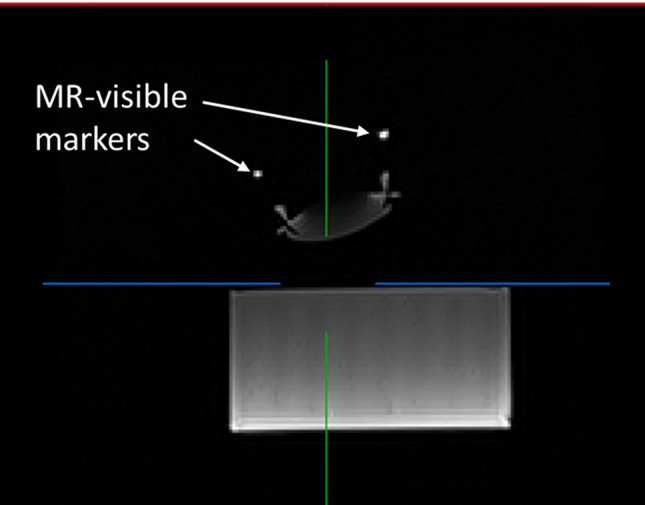
T1-weighted MR image used for the estimation of the transducer position and orientation in the MR scanner.

The experimental protocol can be summarized as follows:

1. A transducer pose is chosen and saved as the desired pose.
2. The user positions the transducer using the navigation system. The supporting legs are rigidified once the user considers that the transducer is at the planned position. An MR loop coil is positioned on top of the transducer to increase the MR signal originating from MR-visible markers (see next step). The MR table is sled to the isocenter of the MRI.
3. A multi-slice, T1-weighted MR dataset is acquired, in which the target and the MR-visible markers contained in the transducer shell are visible (cf. Fig. 5). These markers have a known geometrical configuration with respect to the transducer. Therefore, knowing the 3D positions of these markers in the MR scanner frame allows computing the actual 3D pose (position and orientation) of the transducer in this frame^34^.
4. Finally, since the desired pose is already expressed in the MR reference frame, the positioning error is computed as the distance between the desired and the actual 3D positions of the focus, and the orientation error is computed as the solid angle between the desired and the actual transducer main axis. Details about error calculation are provided in Supplementary Material C.

### Preliminary clinical feasibility demonstration

A first-in-man experiment is reported here. This case is part of an ongoing clinical trial (ClinicalTrials.gov Identifier: NCT04803773) that aims at evaluating the proposed device for treating musculoskeletal tumors. Informed consent was obtained from all participants, or, if participants are under 18, from a parent and/or legal guardian. All methods were carried out in accordance with relevant guidelines and regulations. This study was approved by the French National Regulatory Institution (Agence Nationale de sécurité du médicament et des produits de Santé) and by the Ethics committee (Comité de Protection des Personnes) under Reference 2020-A00306-33.

#### Clinical case

**Figure 6:**
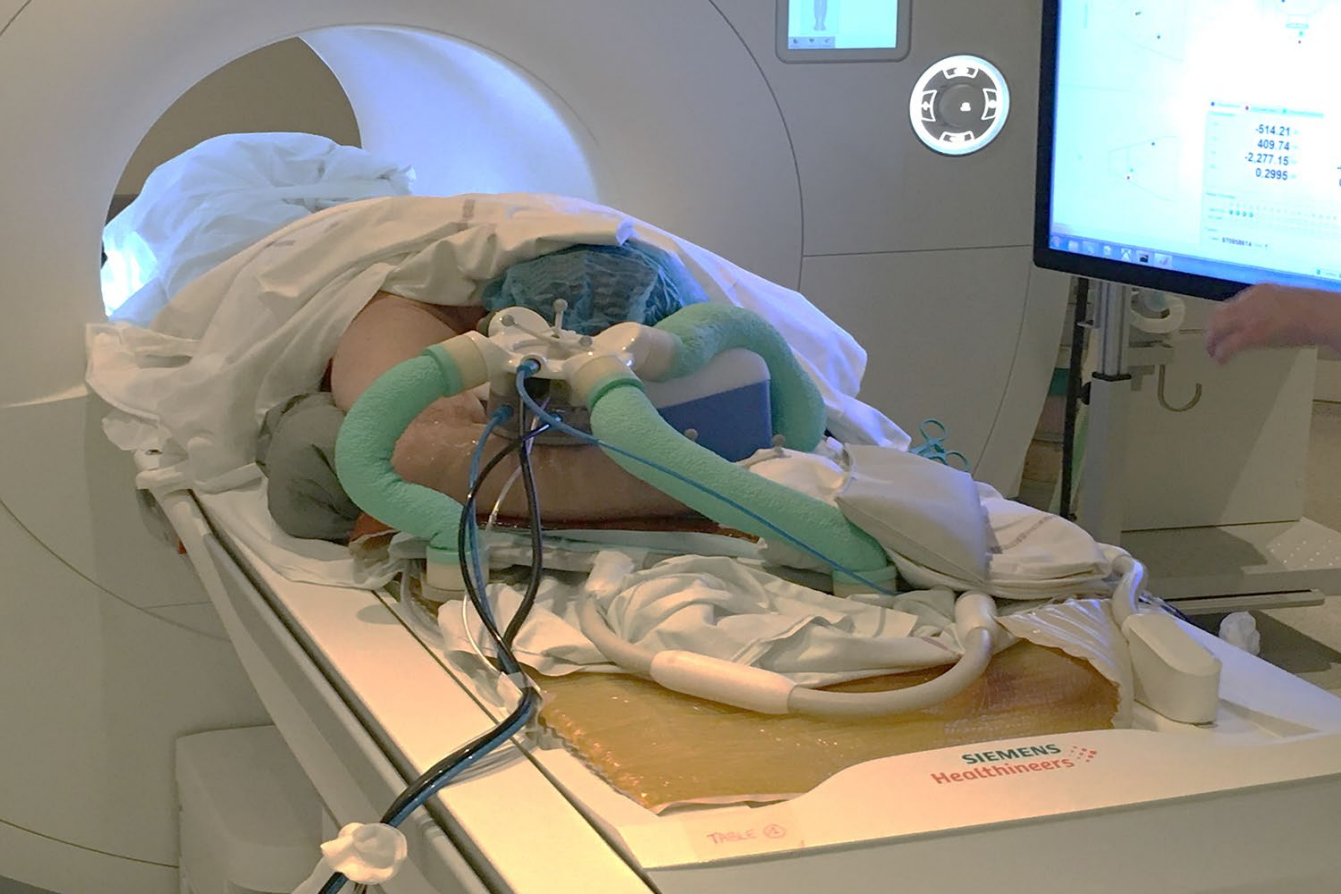
Experimental setup used for the clinical case reported in this study. The transducer is positioned over the forearm, the patient is in a prone position. Only three supporting legs were necessary here. Two feet were ballasted with sandbags, whereas the third was placed under the patient’s arm.

A patient with a painful bone metastasis of a lung adenocarcinoma located in the right forearm was considered for palliative treatment with HIFU. The patient presented mixed inflammatory and mechanical pain, evaluated at 7/10 on the Visual Analog Scale (VAS). The patient also presented a motor deficit of prono-supination evaluated at 3/5. He had no sensory deficit. The bone metastasis was osteolytic, developed on the ulna and invaded the interosseous membrane. This metastasis was an ellipsoid with dimensions approximately equal to 81 mm × 47 mm × 34 mm, the long axis of this ellipsoid coinciding with the long axis of the forearm. The intervention was performed under general anesthesia with the patient in a prone position with the right arm above the head (Fig. 6).

#### Transducer

A 128-element spherical transducer (Imasonic, Voray sur l’Ognon, France) was used for this study. Its central frequency was 1 MHz, and its diameter and nominal focal size were both equal to 60 mm. The transducer is positioned over the right forearm, which was shaved prior to the intervention. Only three supporting legs were necessary to support the transducer. Two feet were ballasted with sandbags, whereas the third was placed under the patient’s arm.

#### HIFU ablation

The planning, transducer positioning, and execution of the therapy was performed by interventional radiologists. Ablation was primarily performed at the periosteum/bone interface, which is generally the most pain-producing site. Transducer positions were carefully chosen to avoid any risk of heating critical nearby structures such as the median and radial nerve. The lesion was covered using 5 different positions of the transducer along the long axis of the lesion. For each transducer position, 3–5 HIFU shots were applied at different foci, using the electronic steering abilities of the transducer. Maximum axial and lateral steering ranges were 20 mm and 5 mm from the natural focus, respectively. The whole lesion was covered in a total of 32 HIFU shots. Each shot had an acoustic power of approximately 50 W. Shot duration was between 16 and 30 s. MRI was performed on a 1.5 T system (MAGNETOM Sola, Siemens Healthineers, Erlangen, Germany). Preoperative MRI consisted in T2-weighted turbo spin echo acquisitions (2 × 2 × 2 mm^3^ isotropic voxels, 10 slices, TR/TE = 3400/77 ms, FOV 349 mm × 399 mm). During the ablation, MR Thermometry was performed using a multislice (N = 5 slices) gradient-recalled MR pulse sequence (TR/TE = 19/12 ms, flip angle 15°, FOV 256 mm × 256 mm, acquisition matrix 115 × 128). Post-operative imaging consisted in T1-weighted Gd-enhanced acquisitions (80 slices, voxel size 0.92 mm × 0.92 mm × 2.5 mm, TR/TE = 7.1/2.4 ms, FOV 285 mm × 380 mm).

## Results

### Mechanical robustness and targeting accuracy

#### Mechanical robustness

Before performing the test on the holding system, the intrinsic drift of the measurement stereo vision system was evaluated to be approximately equal to 0.2 mm in position and 5 × 10^−2^ degrees in orientation after 45 min (cf. gray line in Fig. S2 (a) for position and (b) for orientation).

For the two configurations with the 2 kg-load (Fig. S1), the angular drift was found to reach 0.1° and 0.2° for strongly inclined and arc-like configurations respectively after 45 min (Fig. S2b). Positioning drift was found to be equal to 0.3 mm and 0.2 mm for the strongly inclined and arc-like configurations, respectively (Fig. S2a). These values are comparable with the position computation error given by the stereo vision system which is—constantly—around 0.28 mm for that camera-to-tracker configuration. In light of these results and also considering that the measurement system exhibits an intrinsic drift approximately equal to 0.2 mm in position and 5 × 10^−2^ degrees in orientation, the transducer can be considered as steady under these stress conditions.

#### Targeting accuracy

For each case, the planned pose was compared to the pose measured using the three MRI markers embedded in the cap. Both translation and rotation errors were computed. The average 3D positioning and orientation errors were 5.4 ± 2.2 mm and 4.7° ± 2°, respectively. The positioning duration was between 20 and 76 s for each pose. Full results on 3D errors are provided in Table 1.

**Table 1:** Positioning errors on each axis and 3D Euclidean error. More details on error calculation are provided in Supplementary Material C. Axis (z) corresponds to the central axis of the ultrasonic cone, oriented positively towards the transducer.

Test no	1	2	3	4	5	6	7	8	9	10
Error along x (mm)	4.03	6.54	3.44	2.05	6.69	1.43	4.33	2.80	2.54	− 2.23
Error along y (mm)	− 2.87	− 5.60	− 0.88	2.71	0.90	1.53	− 3.79	2.23	0.73	1.95
Error along z (mm)	0.90	− 6.70	− 3.62	− 0.40	5.45	− 1.62	− 2.18	0.54	0.25	0.16
Error 3D (mm)	4.14	10.59	4.66	4.98	8.56	3.16	6.02	5.01	3.82	3.95

#### Clinical case report

**Figure 7:**
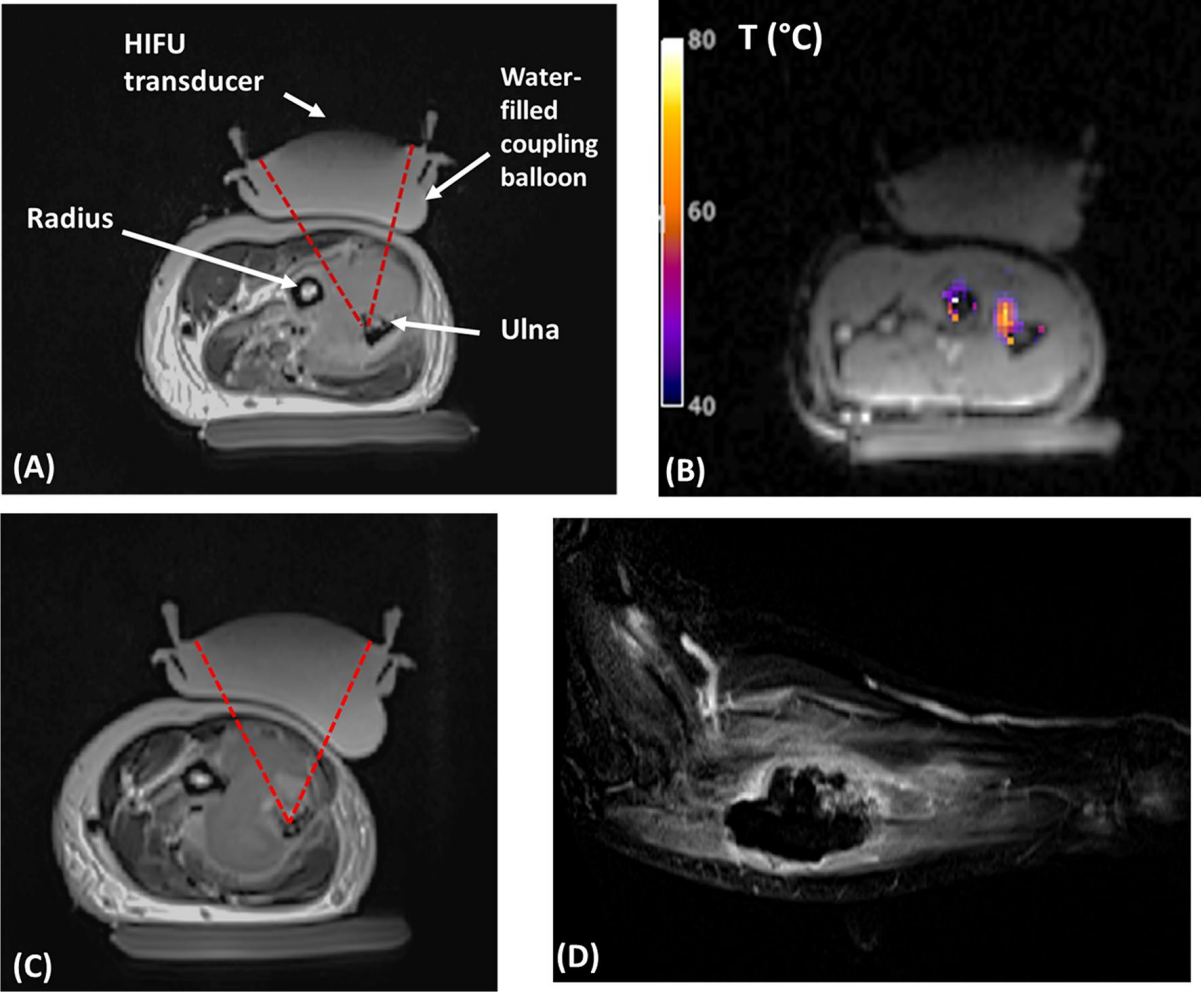
(**A**) Pre-operative T2w anatomical image of the forearm before the first HIFU ablation, illustrating the experimental setup, and showing the metastasis of the ulna. (**B**) Temperature map measured by MR thermometry, showing the temperature elevation in the upper part of the ulna, as planned. (**C**) Intermediate anatomical image after transducer repositioning, at a different MR slice. (**D**) Post-operative Gd-enhanced T1w image, showing in hyposignal the large volume that has undergone necrosis after HIFU ablation.

All HIFU shots were properly executed, all of them yielding a relative temperature elevation of at least 33° (starting from 37°) at the intended target. All shots were of 30 s duration, except for two cases that needed to be interrupted (16 s and 20 s duration) because of high temperature elevation near the skin, assessed by the practitioners participating in this study. Post-operative imaging revealed that approximately 80% of the lesion was necrotic. Figure 7 displays the anatomical preoperative image of the lesion, an example of an MR temperature map obtained during the HIFU ablation, as well as the post-operative Gd-enhanced T1-weighted image.

#### Clinical outcome

The first 2 days after treatment, the patient showed a slight increase in pain due to the oedema, measured at 8/10 on the visual analog pain scale (VAS). The patient showed significant relief of pain starting 3 days after the intervention, with a VAS of 3/10. The reduction in pain persisted during the 3 months of patient follow-up. The inflammatory pain had completely disappeared. The prono-supination deficit remained stable at 3/5. There was still mechanical pain when mobilizing the arm due to bone fragility of the ulna caused by the invasion of the metastasis. No consolidation was performed afterwards. This palliative treatment provided a clear relief of the inflammatory pain. There were no complications nor side effects of the treatment during the follow-up.

## Discussion

This paper introduces a new MR-guided HIFU device whose main feature consists in positioning the HIFU transducer directly on the patient. This system is intended to be modular and flexible for transducer positioning and can also accommodate different transducers with adapted transducer shells and holding platforms. It can hence adapt to a variety of clinical applications that may require different HIFU transducers in terms of central frequency, aperture, number of elements and geometry.

“On-patient” transducer approaches have already been proposed^29^, highlighting the advantages in terms of patient comfort and access to different anatomical locations compared to “In-Table” body systems. However, compared to body systems, one of the major difficulties of “on-patient” approaches is the positioning of the transducer. Our system includes transducer-positioning assistance consisting in a semi-flexible holding device coupled with a virtual reality interface that guides the physician. The granular-jamming-based positioning system allows full flexibility, 6 degrees-of-freedom positioning in its flexible state and rigid bearing of the transducer in its rigid state, while guaranteeing visual contact of the membrane/skin interface at all times thanks to its particular architecture. This architecture renders our device highly modular: the user can choose the number (3 or 4) and the length of the supporting legs (example: long legs for pelvis to surround the patient, short legs for peripheral members), and can adapt the transducer-holding platform connected to the legs to use different transducers for different applications, if needed.

The positioning system was shown to be highly robust. With loading charges of 2 kg (more than twice the weight of the transducer filled with water used in this study), a maximum drift of 0.3 mm was measured after 45 min. This drift falls within the measurement error and can be assumed to be close to zero in real conditions, where some part of the weight is supported by the patient. The whole positioning protocol was also shown to be accurate: the user was able to replicate the planned pose with an overall positioning error of 5.4 mm in terms of focal spot position and 4.7° in orientation. Although such errors are non-negligible, they account for the overall errors committed during the positioning process, including possible manipulation errors during the different steps. For example, a surface MR loop coil was placed directly on the transducer (after having positioned and blocked the system) and may have led to a slight translation in certain configurations, due to the additional weight of the coil on the system. In any case, such error values can be corrected by adjusting the electronic steering of a multi-element transducer such as the one used in this study, if the hottest spot lies a few millimeters away from the target.

Preliminary clinical feasibility was demonstrated in one patient suffering from a bone metastasis located in the forearm. The objective of this first-in-man experience is limited to a clinical feasibility demonstration. No valid medical conclusion in terms of treatment efficiency could be drawn from a single clinical case. All targeted zones were successfully heated to the desired temperature. The clinical outcome was very satisfactory: no adverse effect was reported, and the patient reported a significant and durable decrease of pain level from 7/10 before treatment to 3/10 three days after treatment. The first 2 days after the treatment, the patient presented a slight increase in pain related to the edema created by the HIFU ablation (commonly observed as well with other ablation techniques such as cryoablation, microwave or radiofrequency). Overall, from patient installation to patient evacuation of the MRI room, the whole procedure including preoperative and post-operative imaging lasted 180 min, which was considered as satisfactory for such an extended lesion and considering this was the first clinical case. The use of a 1 MHz, 6 cm focal length transducer was adapted to the relatively superficial nature of the lesion, which was located in the forearm. A less focused, lower frequency transducer can be used for deeper lesions if needed. This highlights one of the major strengths of the proposed device, namely, the fact that the transducer can be easily changed depending on the clinical indication.

The major limitation of the current system is that it is not motorized. Therefore, mechanical transducer trajectories aiming at covering an extended volume are not feasible. To achieve this objective, the presented device requires the combination between electronic steering, limited to a few mm^3^ around the natural focus, and the use of different transducer positions, as illustrated in the reported clinical case. In its current version, our device is therefore limited to relatively small lesions. Alternatively, its versatility makes it particularly relevant for treating multiple, relatively small lesions located in different body parts, such as oligometastatic lesions.

In conclusion, we developed a modular MRgHIFU device that allows positioning the HIFU transducer directly next to the body part that needs to be treated, using a navigation-based guidance. It does not rely on the use of a specific MR system and can be adapted for multiple transducers targeting specific body parts. We expect this device to increase the accessibility to promising MRgHIFU therapies thanks to its versatility and its intrinsic low-cost nature.

## Supplementary Information

Supplementary Information.

## Data Availability

The datasets used and analyzed during the current study available from the corresponding author upon reasonable request.
