# Supplementary material for: A new versatile MR-guided high-intensity focused ultrasound (HIFU) device for the treatment of musculoskeletal tumors
Source: Sci Rep. 2022 May 31;12:9095. doi: 10.1038/s41598-022-13213-1 (PMC9156664; doi:10.1038/s41598-022-13213-1)
Supplement: Supplementary file 1 — Supplementary Information. [file 41598_2022_13213_MOESM1_ESM.docx]

# Supplementary Material

A new versatile MR-guided High-Intensity Focused Ultrasound (HIFU) device for the treatment of musculoskeletal tumors

*Paolo Cabras ^1,2^, Pierre Auloge^3^, Fabrice Bing^1,4^, Pramod Prabakhar Rao^1,3^, Stéphanie Hoarau^2^, Erik Dumont^2^, Alexandre Durand^5^, Benjamin Maurin^5^, Benoit Wach^1^, Loïc Cuvillon^1^, Elodie Breton^1^, Afshin Gangi^1,3^, and Jonathan Vappou^1^*

^1^ICube, Université de Strasbourg, CNRS, UMR 7357, Strasbourg, France

^2^Image Guided Therapy, Pessac, France

^3^Department of Interventional Imaging, Hôpitaux Universitaires de Strasbourg, Strasbourg, France

^4^Radiology Department, Hôpital d’Annecy, Metz-Tessy, France

^5^Axilum Robotics, Schiltigheim, France

Corresponding author: [jvappou@unistra.fr](mailto:jvappou@unistra.fr)

1. System robustness assessment


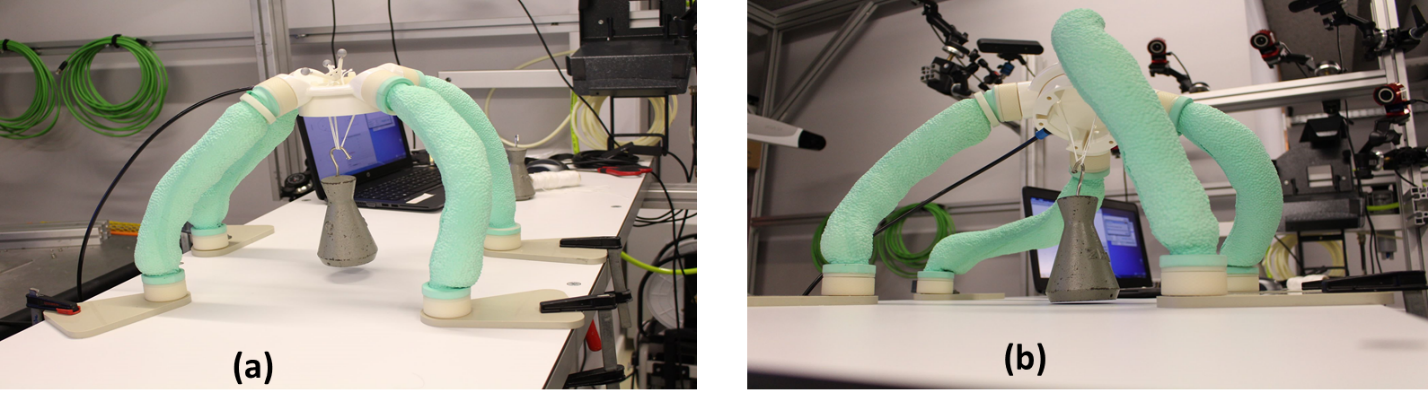


Fig S1: Experimental setup for assessment of mechanical robustness: A 2 kg load is attached to the supporting structure. Two configurations are tested, namely, arc-like (a), and strongly inclined (b). The load does not touch the table.


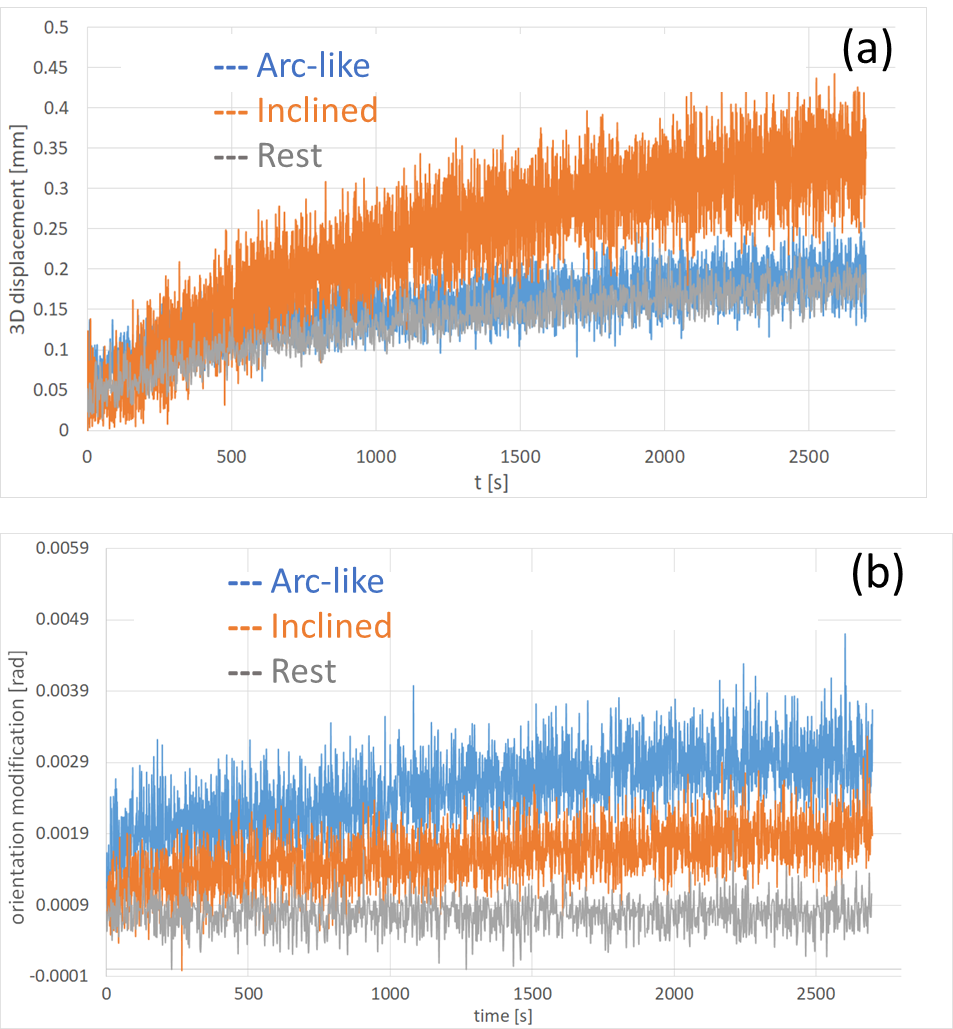


Fig S2: Position (a) and Orientation (b) of the holding system over time (45 mins) for three different configurations: ‘arc configuration’ (cf. Fig S1 (a)) in blue, ‘strongly inclined configuration’ (cf. Fig S1 (b)) in orange. Gray curves correspond to the baseline drift, measured by letting the system ‘at rest’ over the table, without any load.

1. Registration process

# **
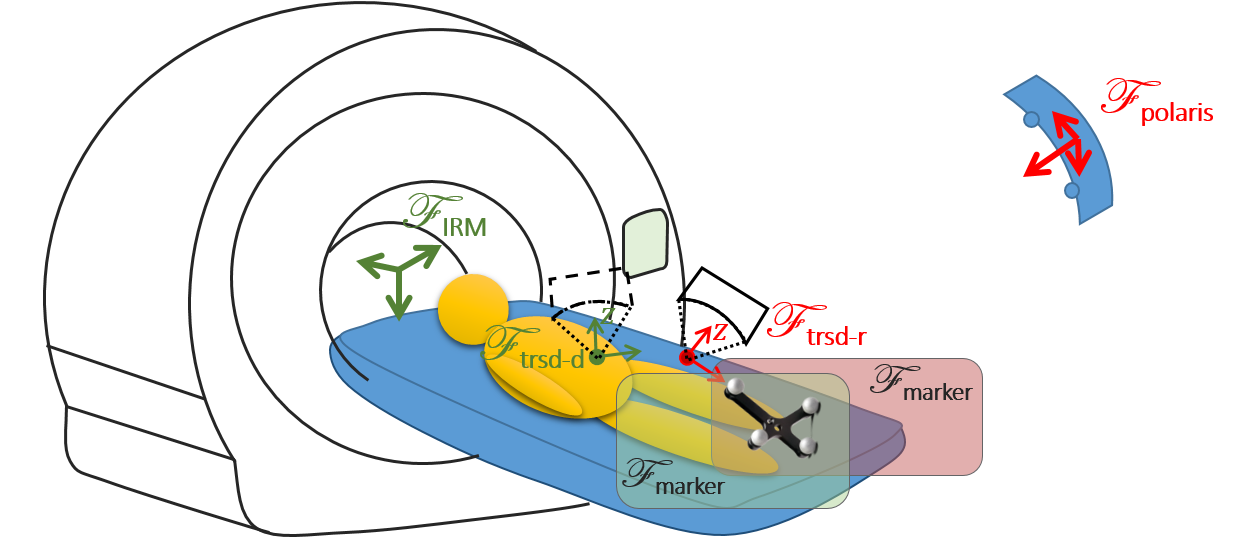
**

Fig S3: Schematic view of the different reference frames involved in the registration process.

During the registration process, the aim is to find the transform between the Polaris reference system (with respect to whom the transducer actual pose is measured - *“trsd-r”*) and the MR reference system (with respect to whom the treatment target and the transducer desired - *“trsd-d”* - pose are defined). Colors in figure S3 are used to help identify the different reference frames.

The hybrid tracker is composed of four spheres visible in both modalities. This element provides the missing link between the two measurement systems (MR and Polaris) allowing computing the desired transform.

${{}^{MR}T}_{trsd-r}= \left[ {{}^{P}T}_{MR} \right]^{-1}{{}^{P}T}_{trsd-r}$ where ${{}^{P}T}_{MR}$ can be computed as

${{}^{P}T}_{MR}= {{}^{P}T}_{markers-P} {{}^{markers-P}T}_{markers-MR} \left[ {{}^{MR}T}_{markers-MR} \right]^{-1}$.

Where:

${{}^{P}T}_{trsd-r}$: is known. It is the current pose of the transducer measured and returned by the Polaris system.

${{}^{P}T}_{markers-P}$: is known. It is the pose of the hybrid tracker (IR reflective sphere) given by the Polaris system.

${{}^{MR}T}_{markers-MR}$: is computed (during registration process). The pose of the hybrid tracker with respect to MRI can be computed fitting the tracker model to the MR landmarks.

${{}^{markers-P}T}_{markers-MR}$: is known. Given the particular design of the hybrid tracker, this transform expressing the spatial relationship between the MR visible markers and the Polaris visible marker is the identity.

1. Methods for error calculation

The 3d error $E$for each tested pose is the 2-norm of the difference vector between the desired $X^{d}$and the measured $X^{m}$ position:

$$E=\left\| X^{d}-X^{m} \right\|_{2}$$

The angular error is the angle difference between the rotation matrices (desired vs measured) , computed as follows:

1. First the difference rotation matrix is computed as $R=R_{d}^{T}R_{m}$(where *d* and *m* are referring to desired and measured respectively).
2. The rotation expressed by $R$is then expressed as axis-angle rotation where the angle θ can be retrieved according to the formula: Trace($R$) = 1 + 2cos(θ)

This gives the absolute angle of a rotation matrix. It is the minimum angle needed to rotate from the identity matrix to$R$, and hence it is a useful pseudo-norm for 3D rotations.
